# 13th European Headache Federation Congress 2019: Meeting Abstracts - part 2: Athens, Greece. May 30–June 12,2019

**DOI:** 10.1186/s10194-020-01092-8

**Published:** 2020-05-12

## A.56 Efficacy of fremanezumab in patients with migraine and documented inadequate response to 2, 3, or 4 classes of migraine preventive treatments: results of the international, multicenter, randomised, placebo-controlled FOCUS study

### Ladislav Pazdera^1^, Xiaoping Ning^2^, Maja Galic^3^, Joshua M. Cohen^2^, Ronghua Yang^2^

#### ^1^Vestra clinics, Rychnov nad Kněžnou, Czech Republic; ^2^Teva pharmaceuticals industries, Frazer, PA, USA; ^3^Teva pharmaceuticals, Amsterdam, the Netherlands

**Background:** Fremanezumab, a fully-humanised monoclonal antibody (IgG2Δa) that selectively targets calcitonin gene-related peptide (CGRP), has proven efficacy for migraine preventive treatment in adults. The FOCUS study of fremanezumab was the first and largest study of a migraine preventive treatment in adults with both chronic and episodic migraine (CM and EM) and documented inadequate response to 2–4 classes of migraine preventive medications.

**Methods:** For 12 weeks of double-blind treatment, patients were randomised (1:1:1) to monthly fremanezumab (Month 1: CM, 675 mg; EM, 225 mg; Months 2 and 3: 225 mg), quarterly fremanezumab (Month 1: 675 mg; Months 2 and 3: placebo), or matched monthly placebo. Changes from baseline in monthly average migraine days and response rates (≥50% reduction in mean monthly number of migraine days for 12 weeks) were evaluated by number of classes of migraine preventive treatments to which patients showed inadequate response.

**Results:** Of 838 randomized patients, 50%, 32%, and 18% had inadequate response to 2, 3, and 4 preventive medication classes, respectively. Changes from baseline in monthly average migraine days over 12 weeks were significantly greater with monthly and quarterly fremanezumab, respectively, vs placebo among patients with inadequate response to 2 (LSMD vs placebo: − 3.7, − 2.9), 3 (− 2.9, − 3.3), or 4 (− 5.4, − 5.3) medication classes (all *P* < 0.0001). Proportions of patients who achieved ≥50% reductions in migraine days at 12 weeks were significantly greater with monthly and quarterly fremanezumab, respectively, vs placebo among patients with inadequate response to 2 (41% and 39% vs 11%), 3 (28% and 32% vs 7%), or 4 (32% and 27% vs 4%) classes (all *P* ≤ 0.002).

**Discussion:** For migraine patients with documented inadequate response to 2, 3, or 4 classes of migraine preventive medications, reductions in monthly average migraine days and clinically meaningful response rates were significantly greater with fremanezumab vs placebo.

## A.57 Impact of age and sex on efficacy of fremanezumab in patients with migraine and documented inadequate response to 2–4 classes of migraine preventive treatments: results of the international, multicentre, randomised, placebo-controlled FOCUS study

### Antoinette MaassenVanDenBrink^1^, Maja Galic^2^, Joshua M. Cohen^3^, Xiaoping Ning^3^, Ronghua Yang^3^, Verena Ramirez-Campos^3^

#### ^1^Department of internal medicine, division of vascular medicine and pharmacology, Erasmus MC, Rotterdam, the Netherlands; ^2^Teva pharmaceuticals, Amsterdam, the Netherlands; ^3^Teva pharmaceuticals industries, Frazer, PA, USA

**Correspondence:** Joshua M. Cohen

**Background:** The FOCUS study of fremanezumab, a fully-humanised monoclonal antibody (IgG2Δa) that selectively targets calcitonin gene-related peptide (CGRP), was the first and largest study of a migraine preventive treatment in adults with both episodic and chronic migraine (EM and CM) and documented inadequate response to 2–4 classes of preventive treatments.

**Methods:** Patients were randomised (1:1:1) to monthly fremanezumab (Month 1: EM, 225 mg; CM, 675 mg; Months 2 and 3: 225 mg), quarterly fremanezumab (Month 1: 675 mg; Months 2 and 3: placebo), or matched monthly placebo for 12 weeks of double-blind treatment. Changes from baseline in monthly average number of migraine days over 12 weeks were evaluated by sex and age.

**Results:** 838 patients were randomised. Reductions in monthly average migraine days were significantly greater with both fremanezumab dosing regimens vs placebo in subgroups of males, females, and ages 18–45 and > 45 years (all *P* < 0.0001; Table).

**Discussion:** Fremanezumab was efficacious, based on statistically significant reductions in monthly average migraine days vs placebo, in males, females, and those aged ≤45 and > 45 years with inadequate response to 2–4 classes of migraine preventive treatments.

**Table 1:** **(abstract A.57).** Change from baseline in monthly average migraine days during 12-weeks of double-blind treatment by sex and age group^a^

	Placebo	Monthly fremanezumab	Quarterly fremanezumab
Males	(*n* = 46)	(*n* = 45)	(*n* = 47)
LSM (SE) change	−0.4 (0.74)	−4.6 (0.78)	−4.2 (0.73)
LSMD (SE) vs placebo	–	−4.2 (0.91)^b^	− 3.8 (0.87)^b^
Females	(*n* = 232)	(*n* = 238)	(*n* = 229)
LSM (SE) change	−0.6 (0.35)	−3.9 (0.34)	−3.6 (0.35)
LSMD (SE) vs placebo	–	−3.3 (0.39)^b^	− 3.0 (0.39)^b^
Age 18–45 years	(*n* = 120)	(*n* = 128)	(*n* = 125)
LSM (SE) change	−0.8 (0.47)	−4.6 (0.49)	−4.1 (0.48)
LSMD (SE) vs placebo	–	−3.8 (0.51)^b^	− 3.2 (0.51)^b^
Age > 45 years	(*n* = 158)	(*n* = 155)	(*n* = 151)
LSM (SE) change	−0.4 (0.48)	−3.8 (0.47)	−3.6 (0.49)
LSMD (SE) vs placebo	–	−3.4 (0.50)^b^	− 3.2 (0.51)^b^

*LSM* least-squares mean, *LSMD* LSM difference

^a^Modified ITT population (*n* = 837)

^b^*P* < 0.0001 vs placebo

## Α.58 Early onset of response to fremanezumab in patients with migraine and a documented inadequate response to 2–4 classes of migraine preventive treatments: results of the international, multicentre, randomised, placebo-controlled FOCUS study

### Egilius L.H. Spierings^1^, Martina Machkova^2^, Xiaoping Ning^3^, Maja Galic^4^, Joshua M. Cohen^3^, Ronghua Yang^3^

#### ^1^Medvadis Research Corporation, Watertown, MA, USA; ^2^CCR Czech Prague, Prague, Czech Republic; ^3^Teva pharmaceuticals industries, Frazer, PA, USA; ^4^Teva pharmaceuticals, Amsterdam, the Netherlands

**Correspondence:** Joshua M. Cohen

**Background:** Preventive treatments for episodic and chronic migraine (EM and CM) have been associated with slow onset of action. Fremanezumab, a fully-humanised monoclonal antibody (IgG2Δa) that selectively targets calcitonin gene-related peptide (CGRP), has proven efficacy for preventive migraine treatment in adults. The FOCUS study of fremanezumab was the first and largest study of a migraine preventive treatment in adults with both EM and CM and documented inadequate response to 2–4 classes of migraine preventive treatments.

**Methods:** Patients were randomised (1:1:1) to monthly fremanezumab (Month 1: EM, 225 mg; CM, 675 mg; Months 2 and 3: 225 mg), quarterly fremanezumab (Month 1: 675 mg; Months 2 and 3: placebo), or matched monthly placebo for 12 weeks of double-blind treatment. Proportions of responders (≥50% and ≥ 75% reduction in migraine days) over the first 4 weeks were evaluated as secondary and exploratory endpoints, respectively. Changes from baseline in weekly migraine days were compared using a mixed-effects model for repeated measures.

**Results:** 838 patients were randomised. With monthly and quarterly fremanezumab, respectively, vs placebo, significantly higher proportions of patients achieved ≥50% reductions (36% and 38% vs 10%) and ≥ 75% reductions (14% and 14% vs 2%) in migraine days over the first 4 weeks (all *P* < 0.0001). Reductions from baseline in weekly migraine days were significantly greater with fremanezumab (LSM[SE] change: monthly, − 0.9[0.11]; quarterly, − 1.0[0.11]) vs placebo (− 0.1[0.11]) by Week 1 and at each weekly time point through Week 4 (all P < 0.0001).

**Discussion:** Monthly and quarterly fremanezumab demonstrated early onset of efficacy, with significantly greater clinically meaningful response rates after 4 weeks of treatment and significantly greater reductions from baseline in weekly migraine days as early as Week 1 vs placebo, in patients with EM or CM and documented inadequate response to 2–4 classes of migraine preventive treatments.

## Α.59 Efficacy, clinically meaningful responses, and impact on acute headache medication use with fremanezumab in patients with migraine and documented inadequate response to 2–4 classes of migraine preventive treatments: results of the international, multicentre, randomised, placebo-controlled FOCUS study

### Michel D. Ferrari^1^, Hans Christoph Diener^2^, Egilius L.H. Spierings^3^, Xiaoping Ning^4^, Maja Galic^5^, Joshua M. Cohen^4^, Ronghua Yang^4^, Messoud Ashina^6^

#### ^1^Leiden University Medical Centre (LUMC), Leiden, The Netherlands; ^2^Faculty of Medicine, University of Duisburg-Essen, Essen, Germany; ^3^Medvadis Research Corporation, Watertown, MA, USA; ^4^Teva Pharmaceuticals Industries, Frazer, PA, USA; ^5^Teva Pharmaceuticals, Amsterdam, The Netherlands; ^6^Danish headache Centre, Department of Neurology, Rigshospitalet, Glostrup, Denmark

**Correspondence:** Joshua M. Cohen

**Background:** Fremanezumab, a fully-humanised monoclonal antibody (IgG2Δa) that selectively targets calcitonin gene-related peptide (CGRP), has proven efficacy for migraine preventive treatment in adults. The FOCUS study of fremanezumab was the first and largest study of a migraine preventive treatment in adults with both episodic and chronic migraine (EM and CM) and documented inadequate response to 2–4 classes of migraine preventive treatments.

**Methods:** Patients were randomised (1:1:1) to monthly fremanezumab (Month 1: EM, 225 mg; CM, 675 mg; Months 2 and 3: 225 mg), quarterly fremanezumab (Month 1: 675 mg; Months 2 and 3: placebo), or matched monthly placebo for 12 weeks. The primary efficacy endpoint was the change from baseline in monthly average migraine days over 12 weeks. Responder rates (≥50% and ≥ 75% reduction in migraine days) and monthly average days of acute headache medication use were also evaluated.

**Results:** 838 patients were randomised. Reductions from baseline in monthly average migraine days over 12 weeks were greater with fremanezumab (LSM[SE] change: monthly, − 4.1[0.34]; quarterly, − 3.7[0.34]) vs placebo (− 0.6[0.34]; both *P* < 0.0001). With monthly and quarterly fremanezumab, respectively, vs placebo, higher proportions of patients achieved ≥50% (34% and 34% vs 9%) and ≥ 75% (12% and 8% vs 2%) reductions in migraine days over 12 weeks (all *P* ≤ 0.0002). Reductions in monthly average days of acute headache medication use were greater with fremanezumab (LSM[SE] change: monthly, − 3.9[0.32]; quarterly, − 3.7[0.32]) vs placebo (− 0.6[0.32]) over 12 weeks, as were reductions in monthly average days of migraine-specific acute headache medication use (all P < 0.0001).

**Discussion:** Reductions in monthly average migraine days and days of acute headache medication use and clinically meaningful response rates were significantly greater with fremanezumab vs placebo in migraine patients with documented inadequate response to 2–4 classes of migraine preventive treatments.

## A.60 Efficacy and safety of fremanezumab in patients with migraine and documented inadequate response to 2–4 classes of migraine preventive treatments: results of the international, multicentre, randomised, placebo-controlled FOCUS study

### Michel D. Ferrari^1^, Hans Christoph Diener^2^, Xiaoping Ning^3^, Maja Galic^4^, Joshua M. Cohen^3^, Ronghua Yang^3^, Messoud Ashina^5^

#### ^1^Leiden university medical Centre (LUMC), Leiden, the Netherlands; ^2^University of Duisburg-Essen, Essen, Germany; ^3^Teva pharmaceuticals industries, Frazer, PA, USA; ^4^Teva pharmaceuticals, Amsterdam, the Netherlands; ^5^Danish headache Centre, Department of Neurology, Rigshospitalet, Glostrup, Denmark

**Correspondence:** Joshua M. Cohen

**Background:** Fremanezumab, a fully-humanised monoclonal antibody (IgG2Δa) that selectively targets calcitonin gene-related peptide (CGRP), is effective for the preventive treatment of migraine. The FOCUS study of fremanezumab was the first and largest study of a migraine preventive treatment in patients with both episodic and chronic migraine (EM and CM) and documented inadequate response to 2–4 classes of migraine preventive treatments.

**Methods:** For 12 weeks of double-blind treatment, patients were randomised (1:1:1) to monthly fremanezumab (Month 1: EM, 225 mg; CM, 675 mg; Months 2 and 3: 225 mg), quarterly fremanezumab (Month 1, 675 mg; Months 2 and 3, placebo), or matched monthly placebo. The primary efficacy endpoint was mean change from baseline in monthly average migraine days over 12 weeks and was compared using analysis of covariance.

**Results:** 838 patients were randomised. Reductions from baseline in monthly average migraine days over 12 weeks were significantly greater with monthly fremanezumab (least-squares mean[SE] change, − 4.1[0.34]) and quarterly fremanezumab (− 3.7[0.34]) versus placebo (− 0.6[0.34]; both *P* < 0.0001) in the overall population. In subgroups of patients with EM and CM, reductions from baseline in monthly average migraine days were also significantly greater with both fremanezumab regimens versus placebo (all P < 0.0001). The incidence of AEs was similar in the placebo and combined fremanezumab groups, respectively, including overall AEs (48% and 50%), AEs leading to discontinuation (1% and < 1%), and SAEs (1% and 1%). None of the SAEs were considered treatment-related, and no safety signals were identified.

**Discussion:** Fremanezumab demonstrated significant improvements in efficacy, based on reductions in monthly average migraine days versus placebo, and was safe and well tolerated over 3 months in patients with EM or CM and documented inadequate response to multiple classes of migraine preventive treatments.

